# Donor-Derived CD7 CAR-T Therapy Followed by Allogeneic Hematopoietic Stem Cell Transplantation for Acute T-Lymphocytic Leukemia Associated With Hepatitis B: A Case Report

**DOI:** 10.3389/fimmu.2022.931452

**Published:** 2022-07-12

**Authors:** Zhihui Li, Fanqiao Meng, Jing Li, Tong Wu

**Affiliations:** Bone Marrow Transplantation, Beijing Boren Hospital, Beijing, China

**Keywords:** hepatitis B virus, donor-derived chimeric antigen receptor T cells, allogeneic hematopoietic stem cell transplantation, relapsed/refractory acute T-lymphocytic leukemia, stem cell transplantation

## Abstract

The use of chimeric antigen receptor T cells (CAR-Ts) is effective in the treatment of hematological malignancies. It has been reported that HBV is reactivated after CAR-T immunotherapy for refractory/relapsed hematological malignant B-cell tumors. However, there is little literature on donor-derived CAR-T therapy combined with allogeneic hematopoietic stem cell transplantation in hepatitis B patients with acute T-lymphocytic leukemia. We report the case of one patient with hepatitis B associated with relapsed/refractory acute T-lymphocytic leukemia (T-ALL) treated with donor-derived CD7 CAR-T therapy and allogeneic hematopoietic stem cell transplantation. During treatment, the copy number of hepatitis B virus continuously decreased, and AST, ALT, DBIL and TBIL remained within the controllable ranges. CD7-negative MRD recurred 4.5 months after transplantation, and the flow cytometry results became negative after immunosuppressive reduction. Seven months after transplantation, the patient had complete remission, and the copy number of hepatitis B virus decreased to below 10^2^. This is the first study on the safety and effectiveness of donor-derived CD7 CAR-T therapy bridging to allogeneic hematopoietic stem cell transplantation in a patient with relapsed/refractory acute T-lymphocytic leukemia and hepatitis B.

## Introduction

In acute T-lymphocytic leukemia (T-ALL), abnormal proliferation and accumulation of malignant T lymphocytes in the bone marrow inhibits normal hematopoiesis, and malignant T lymphocytes can invade extramedullary tissues, such as the brain, lymph nodes, gonads, and liver, eventually leading to patient death. The treatment of T-ALL is challenging in the clinic (1, 2). Studies have shown that chimeric antigen receptor T-cell (CAR-T) immunotherapy has great potential in the treatment of relapsed/refractory acute B-lymphocytic leukemia (B-ALL), especially for patients who do not achieve complete remission after multiple chemotherapy or radiotherapy courses (3). Relapsed/refractory B-ALL patients who achieved complete remission after CAR-T therapy and then underwent bridging allogeneic hematopoietic stem cell transplantation showed significantly longer survival (4), while CAR-T therapy for T-ALL is rarely reported. Zhou et al. reported on one patient with DLBCL who died of hepatitis after CAR-T therapy, which may be because CAR-T therapy activated the patient’s hepatitis B virus. However, Wang et al. showed that CAR-T therapy had no impact on the safety of hepatitis B patients or the effectiveness of their treatment (4, 5).

Here, we report the case of the first patient with hepatitis B associated with relapsed/refractory acute T-lymphocytic leukemia (T-ALL) treated with donor-derived CD7 CAR-T therapy followed by allogeneic hematopoietic stem cell transplantation. The information provided here can serve as a reference for the application of donor-derived CD7 CAR-T therapy and allogeneic hematopoietic stem cell transplantation in the clinical treatment of T-ALL associated with hepatitis B.

## Case Report

**Figure 1:**
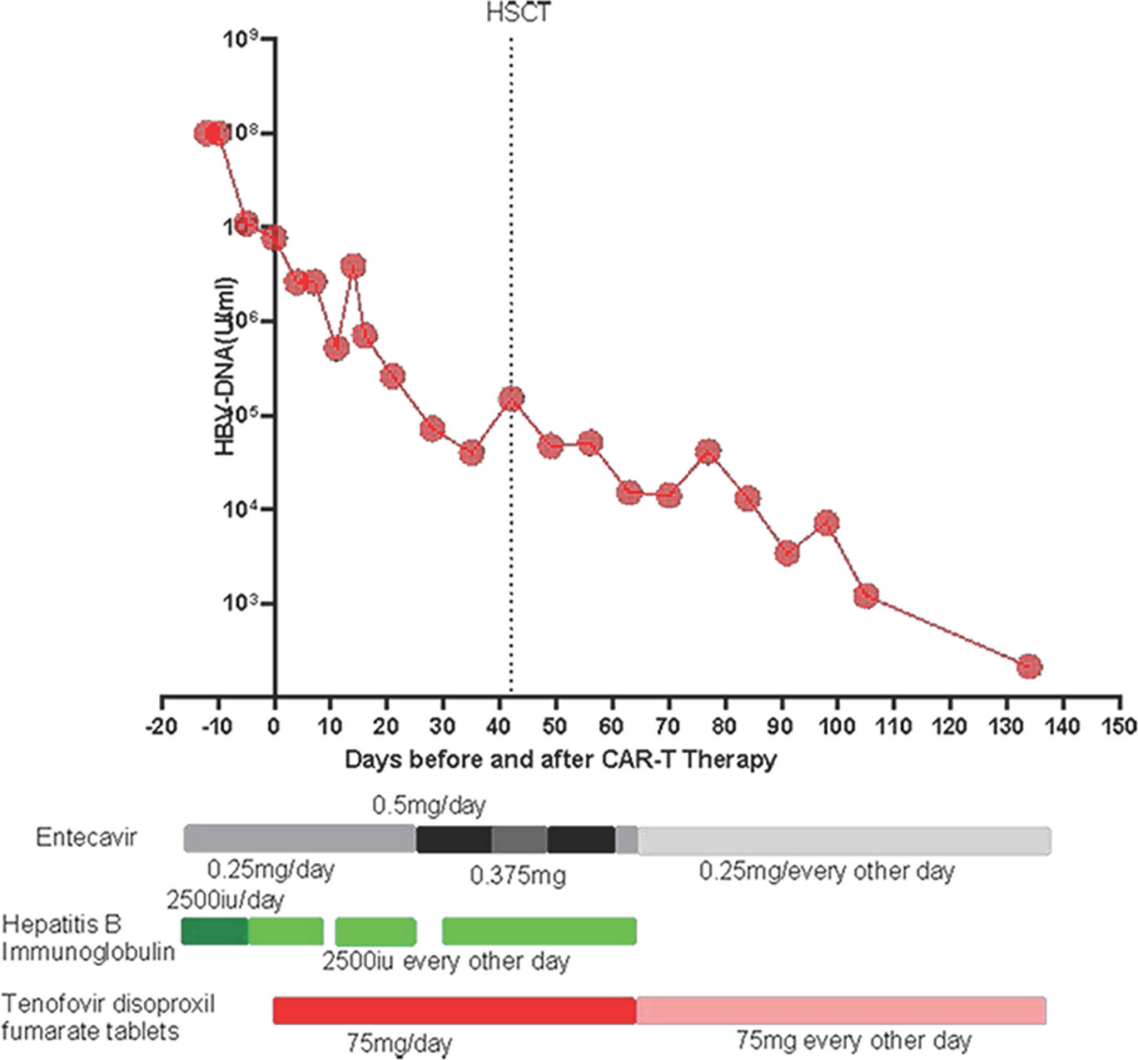
Levels of HBV DNA during CD7 CAR-T therapy bridging to HSCT.

The patient was a 3-year-old girl. In January 2020, because of pale complexion and petechiae over the entire body, she was admitted to a local hospital. She had a full-term natural birth and normal growth and development. Her father was in good health, and her mother had hepatitis B virus infection. They were not in a consanguineous marriage. The parents denied a family history of similar or other cancers or hereditary diseases. Her white blood cell count was 209×10^9/L. She was diagnosed with acute lymphocytic leukemia by MICM of the bone marrow. Since January 15, 2020, according to the CCLG-ALL 2015 protocol, she was evaluated as having intermediate risk and underwent induction chemotherapy. The reexamination showed remission in the bone marrow morphology and MRD<0.01%. After that, she was given regimens of CAM and HD-MTX+6-MP. On August 7, 2020, morphological evaluation of her bone marrow showed 4.5% lymphoblasts and prolymphocytes; flow cytometry indicated 9.85% abnormal naive T lymphocytes. Thus, she was suspected to have a recurrence of acute lymphocytic leukemia. Peripheral blood smears indicated 4% naive T lymphocytes. BLOCK3 chemotherapy was given. On September 18, 2020, morphological evaluation of bone marrow showed 24.5% lymphoblasts and prolymphocytes, and flow cytometry showed 19.98% abnormal naive T lymphocytes. She was given VDS+DEX and VDIP regimens successively, but with no remission. During this period, no abnormal lymphocytes were found in multiple lumbar punctures + sheath injections of cerebrospinal fluid. On November 10, 2020, the patient was admitted to our hospital for the first time. MICM confirmed the previous diagnosis of acute T-lymphocytic leukemia. Gene mutations identified Notch1 p.S2467Pfs * 12, STAT5A p.T6285, and IL7R p.L243_T244insCP, but HBV DNA>1*10^8 IU/ml suggested associated hepatitis B. She was given entecavir 0.25 mg once per day and an intravenous injection of freeze-dried hepatitis B human immunoglobulin 2500 IU once per day for antiviral treatment. The changes in HBV DNA during treatment are illustrated in **Figure 1**. On November 19, 2020, the FLU + idarubicin chemotherapy regimen was given to eliminate lymphocytes; on November 25, 2020, she was transfused with humanized CD7 CAR-T cells from a donor (her father). The donor’s physical examination results were normal, and his HBV DNA was measured at 0. On Days 15 and 30 after CD7 CAR-T therapy (2020-12-15 and 2020-12-24), the bone puncture reexamination showed morphological remission, with residue negativity. The treatment process is outlined in **Table 1**.

**Table 1:** Brief chronology of the key clinical events in this case.

Time before and after CD7 CAR-T therapy	Date	Key events
−10 months	2020.01.15	Diagnosis of T-ALL
−10 months	2020.01.20	PVDL chemotherapy (Pred+VCR+DNR+L-ASP)
−9 months	2020.02.19	CAT chemotherapy (CTX+Ara-C+6-TG)
−8 months to −3 months	2020.03.05- 2020.08.01	1 cycle of CAT+VCR+PEG-ASP, 4 cycles of HD-MTX+6-MP chemotherapy, replese
-110 days	2020.08.07	BLOCK3 chemotherapy, replese
-68 days	2020.09.18	VDS+DEX chemotherapy
-45 days	2020.10.11	VDIP chemotherapy (VDS1+DEX+IDA+PEG-asp)
-6 days	2020.11.19	FLU+MTX+Idarubicin chemotherapy
0 day	2020.11.25	CD7 CAR-T
31 days	2020.12.26	FLU/BU/ATG/Me-CCNU
40 days	2021.01.04	HSCT

**Figure 2:**
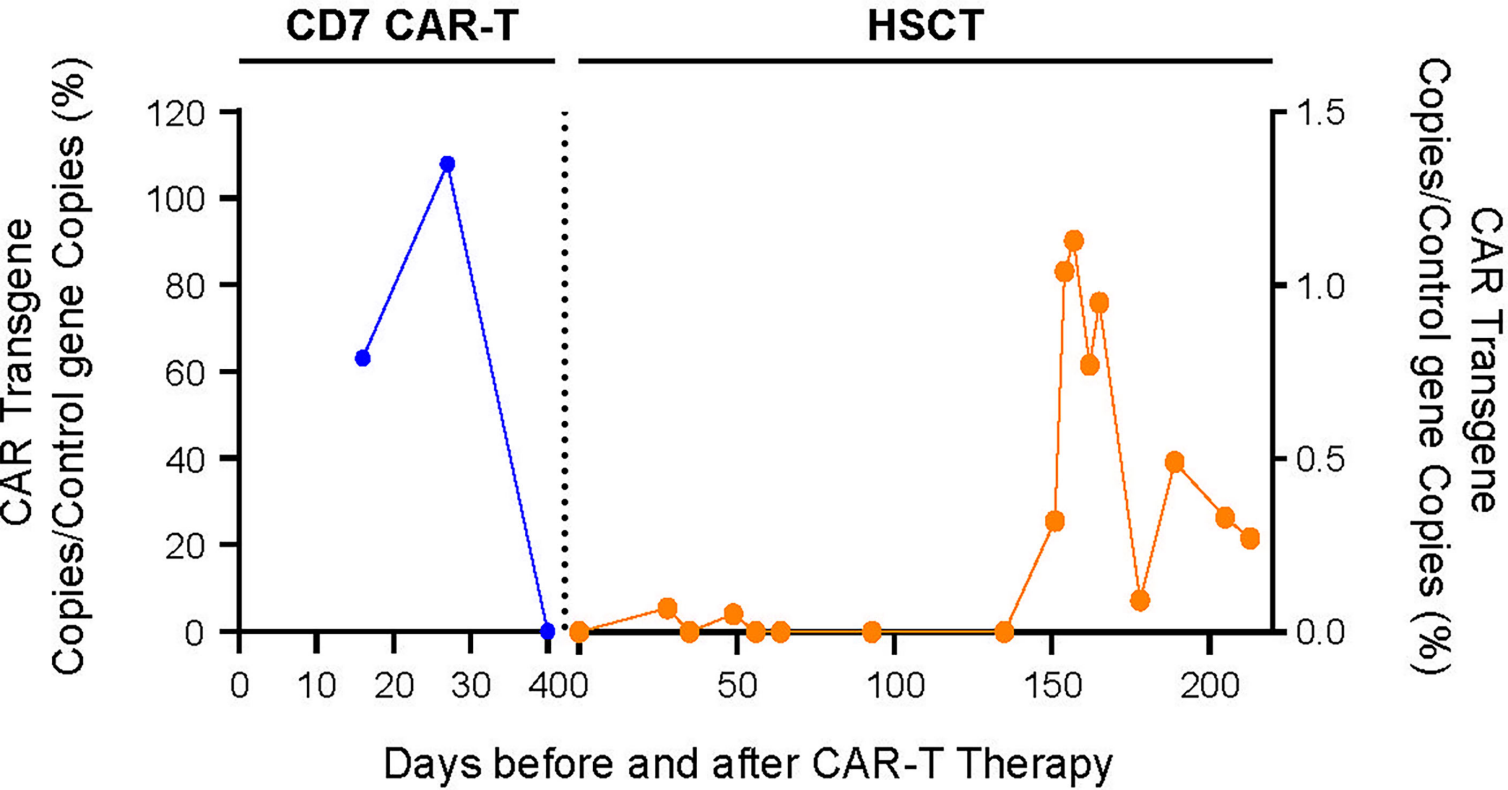
PCR was used to monitor the change trend of CD7 CAR T-cell count *in vivo* during CAR-T and transplantation.

**Figure 3:**
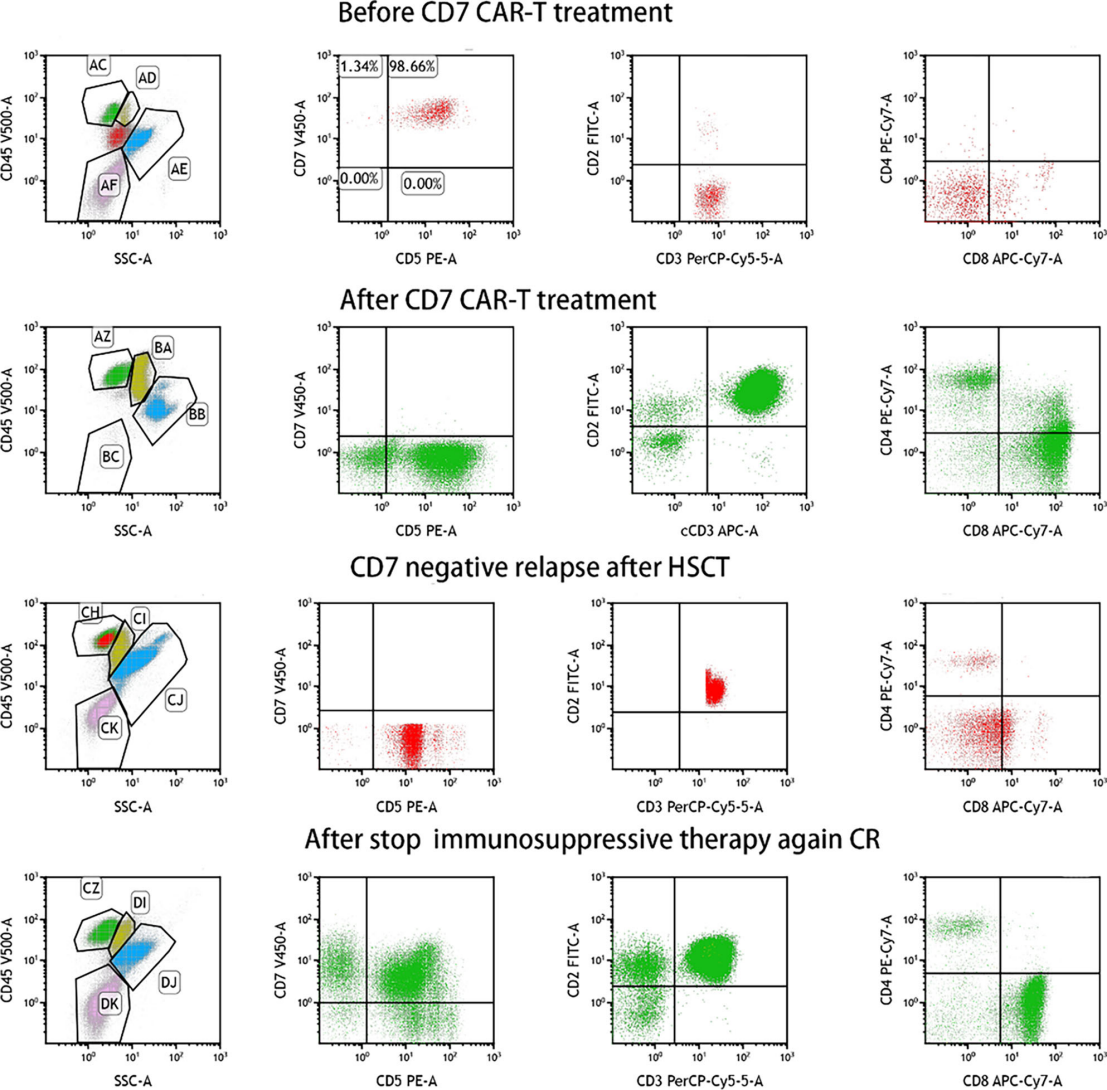
MRD was detected by flow cytometry during CD7 CAR-T therapy bridging to HSCT.

Four days after CAR-T transfusion, a CRS reaction (grade 2 at maximum) occurred, which was relieved after hormone therapy. She had a rash 3 weeks after CAR-T therapy, which was considered aGVHD (skin grade II) and was relieved after intense anti-GVHD treatment. Two weeks after CD7 CAR-T transfusion, the white blood cell count was lower than 0.5×10^9/L. After CAR-T transfusion, she still received anti-hepatitis B virus therapy. The HBV DNA copy number increased after CAR-T transfusion and peaked (3.9*10^6^ IU/ml) on the 14th day. After consultation with a liver disease specialist, since there was the possibility of the activation of the hepatitis B virus, tenofovir disoproxil fumarate tablets were given at a dosage of 75 mg once per day, in addition to entecavir and hepatitis B globulin. After that, her hepatitis B HBV DNA copy number fell to 7.3×10^4 IU/ml before transplantation. The BU/FLU/ATG/Me-CCNU conditioning regimen started on December 26, 2020. The donor of CAR-T was also used for the transplant. She subsequently received HLA 5/10–matched, blood type–mismatched (donor O+, recipient B+) haploidentical stem cell transplantation from her father on January 4, 2021. Graft-versus-host disease (GVHD) prophylaxis was performed with cyclosporine, short-term methotrexate and mycophenolate mofetil. Seven days after transplantation, the child had diarrhea and persistent bloody stools, but no pathogenic bacteria were found in the culture; engraftment syndrome was excluded. Eighteen days after transplantation, the neutrophil count was 0.25×10^9^/L, suggesting that the neutrophil were not engrafting. The patient had gastrointestinal bleeding during the neutropenic period soon after the myeloablative conditioning regimen. The bleeding may have been due to the side effects of the anti-hepatitis B drugs and thrombocytopenia. The bleeding symptoms gradually improved after reducing the doses of tenofovir disoproxil fumarate and entecavir and transfusing platelets. The time of neutrophil engraftment was on days 22 after transplantation. The time of platelets engraftment was on days 91 after transplantation. The plasma CMV DNA and EBV DNA of the patient were all negative after transplantation. Bone marrow puncture and lumbar puncture at 1, 2 and 3 months after transplantation showed that the bone marrow morphology was in remission, with residue negativity, and the chimerism rates of bone marrow blood and peripheral blood showed it was of the complete donor type. After transplantation, PCR detected CD7 CAR-Ts again, and stem cell implantation had no effect on hepatitis B virus replication (**Figure 2**). Four and a half months after transplantation, abnormal T lymphocytes expressing CD7-CD5+ were detected by flow cytometry, but the patient was still in complete remission with full donor chimerism in both bone marrow and CD3+ cells in peripheral blood. Half a month after reducing immunosuppressive agents, the flow cytometry of bone marrow turned negative (**Figure 3**). As of August 2021, eight months after transplantation, the patient showed complete remission and was HBV DNA negative.

**Figure 4:**
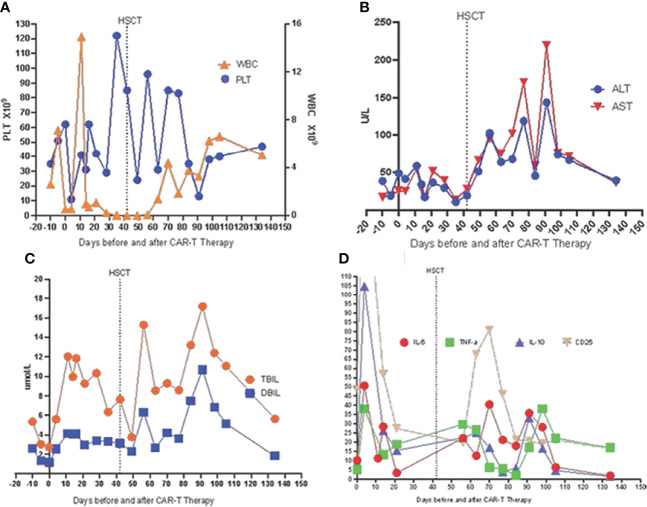
Sequential infusion of anti-CD7 CAR-T therapy bridging to HSCT. **(A)** Levels of WBC and PLT during CAR-T therapy bridging to HSCT. **(B)** Levels of ALS and AST during CAR-T therapy bridging to HSCT. **(C)** Levels of TBIL and DBIL during CAR-T therapy bridging to HSCT. **(D)** Levels of IL-6, TNF-a, IL-10 and CD25 during CAR-T therapy bridging to HSCT.

In this case, the patient reached complete remission after CD7 CAR-T therapy, and then bridging HSCT was performed. HBV DNA increased temporarily after CD7 CAR-T therapy and then gradually decreased. Blood indicators (white blood cells, platelets), biochemical indicators (AST, ALT, DBIL, TBIL), and cytokines (interleukin 6, tumor necrosis factor α, interleukin 10, soluble CD25, interferon γ) were all monitored (**Figure 4**). White blood cells increased to a maximum of 14.93*10^9^/L 11 days after CD7 CAR-T therapy, dropped to less than 0.5*10^9^/L, and remained in continuous agranulocytosis. Engrafted leukocytes survived 18 days after transplantation (**Figure 4A**). The time of neutrophil engraftment was on days 22 after transplantation. Since the patient had hepatitis B virus replication, liver function was monitored (AST and ALT are illustrated in **Figure 4B**, TBIL and DBIL in **Figure 4C**). There was a transient increase in transaminase and bilirubin after transplantation. At that time, there were no other GVHD clinical manifestations such as rash, diarrhea. It has been improved with strengthening liver protection medicines without addition of anti-GVHD treatment. Therefore, the diagnosis of abnormal liver function was considered to be drug-induced liver damage rather than GVHD. The concentrations of cytokines (interleukin 6, tumor necrosis factor α, interleukin 10, soluble CD25, interferon γ) peaked four days after CD7 CAR-T treatment, consistent with the CRS response, then increased to another peak after HSCT, and fell after that (**Figure 4D**).

## Discussion

Immunotherapy with chimeric antigen receptor cells has been widely applied in the treatment of B-cell hematological malignancies (6, 7). It is also used for the treatment of T-cell malignancies. At present, the clinical application of CAR-T therapy is gradually being expanded and improved. During CAR-T therapy, the treatment of other, associated diseases needs to be considered. Many HBV patients are excluded from CAR-T therapy. Patients who have eliminated their HBV infection are still at risk of HBV reactivation.

HBV is a double-stranded DNA virus that can induce host immune responses in MHC II-CD4+ helper T cells and MHC I-CD8+ cytotoxic T cells in liver cells. HBV reactivation is observed in immunosuppressive HBsAg-positive patients (8), especially when immunosuppressive therapy is combined with chemotherapy with cyclophosphamide, doxorubicin, vincristine and prednisone (9–11). Clinical guidelines recommend that antiviral prophylaxis be given during chemotherapy and at least 12 to 24 months after the termination of the immunosuppressive regimen (12–14). Currently, there is no consensus or guideline for the prevention of HBV reactivation in patients treated with CAR-T therapy, but long-term prevention is still necessary. The monitoring of HBV DNA, including testing for HBsAg, anti-HBcAb and anti-HBs, is helpful for screening patients receiving CAR-T therapy for hepatitis B virus activation during the clinical process. It has been reported that continuous infusion of CD19 and CD22 CAR-Ts can prolong B-cell hypoplasia and T-cell immune reconstruction, so antiviral prevention should last for more than 12 months to prevent HBV reactivation (5, 15). Cao et al. (16) recommended that patients with suspected hepatitis B or confirmed progression of hepatitis B take tenofovir instead. Yang et al. (17) reported HBV reactivation after drug withdrawal (tenofovir) during CAR-T therapy, which was also controlled after the therapy was restarted. However, in the study of Wei et al (5), the readministration of entecavir was ineffective for reactivated HBV, and the patient died of liver function deterioration in hepatic coma. Lai et al. (18) found that even CAR-T cells from HBV-infected patients showed strong antitumor potential.

In our study, for the first time, donor-derived CD7 CAR-T cells were used in the treatment of relapsed/refractory T-ALL, achieving good efficacy (remission rate: 83%). With regard to safety, most adverse reactions occurred within 30 days of CD7 CAR-T transfusion, including CRS, GVHD, cytopenia, infection and virus activation, and these adverse reactions were controllable, being relieved after symptomatic treatment, which demonstrates the effectiveness and safety of donor-derived CD7 CAR-T therapy for relapsed/refractory T-ALL, giving it great potential for use in the clinic (19). During donor-derived CD7 CAR-T therapy, the patient is in immunosuppression and immunodeficiency, which necessitates a long process for immune reconstitution. In previous clinical trials, HBV carriers were advised to keep taking prophylactic antiviral therapy for at least six months after their B cells had fully recovered. To date, our antiviral treatment has lasted for 9 months, and the patient is in good general condition, with HBV DNA <10^2^ IU/ml. In this case, after being treated with donor-derived CD7 CAR-Ts, the patient had transient hepatitis B virus activation on Day 14 after CD7 CAR-T transfusion, but the HBV DNA quickly decreased to a safe level, indicating that donor-derived CD7 CAR-Ts cannot activate the hepatitis B virus. During subsequent transplantation, anti-hepatitis B drugs may have had an impact on leukocyte engraftment and caused intestinal bleeding. Therefore, we reduced the doses of anti-hepatitis B drugs (entecavir and tenofovir), but HBV DNA did not increase, and leukocytes survived. CD7 CAR-T cells were observed again after transplantation, with no effect on the replication of HBV. Therefore, donor-derived CD7 CAR-T therapy followed by allogeneic hematopoietic stem cell transplantation is safe in the treatment of T-ALL associated with hepatitis B. Since this study was only a case report, further research is needed to verify this conclusion.

This is the first report on donor-derived CD7 CAR-T therapy bridging to allogeneic hematopoietic stem cell transplantation in the treatment of T-ALL associated with hepatitis B. More data are needed to evaluate HBV replication, and the patient should be followed up to assess the duration of preventive anti-HBV DNA therapy after transplantation.

## Data Availability Statement

The original contributions presented in the study are included in the article/supplementary material. Further inquiries can be directed to the corresponding author.

## Ethics Statement

This article was approved by the ethical committee of Beijing Boren Hospital (ChiCTR2000040641). The patients/participants provided their written informed consent to participate in this study.

## Author Contributions

ZL wrote the article and collected the data. TW made the treatment plan and reviewed the final paper. FM and JL analyzed the data. All authors contributed to the article and approved the submitted version.

## Conflict of Interest

The authors declare that the research was conducted in the absence of any commercial or financial relationships that could be construed as a potential conflict of interest.

## Publisher’s Note

All claims expressed in this article are solely those of the authors and do not necessarily represent those of their affiliated organizations, or those of the publisher, the editors and the reviewers. Any product that may be evaluated in this article, or claim that may be made by its manufacturer, is not guaranteed or endorsed by the publisher.
